# Serum metabolomics detected by LDI‐TOF‐MS can be used to distinguish between diabetic patients with and without diabetic kidney disease

**DOI:** 10.1002/2211-5463.13683

**Published:** 2023-08-11

**Authors:** Fengmei Qian, Li Zhao, Di Zhang, Mengjie Yu, Wei Zhou, Juan Jin

**Affiliations:** ^1^ The Second School of Clinical Medicine Zhejiang Chinese Medical University Hangzhou China; ^2^ Department of Nephrology, Urology & Nephrology Center Zhejiang Provincial People's Hospital (Affiliated People's Hospital, Hangzhou Medical College) China; ^3^ Department of Nephrology The First People's Hospital of Hangzhou Lin'an District, Affiliated Lin'an People's Hospital, Hangzhou Medical College China

**Keywords:** diabetes, diabetic kidney disease, LDI‐TOF‐MS, machine learning, serum metabolomics

## Abstract

Diabetic kidney disease (DKD) is an important cause of end‐stage renal disease with changes in metabolic characteristics. The objective of this study was to study changes in serum metabolic characteristics in patients with DKD and to examine metabolite panels to distinguish DKD from diabetes with matrix‐assisted laser desorption/ionization time‐of‐flight mass spectrometry

(MALDI‐TOF‐MS). We recruited 40 type II diabetes mellitus (T2DM) patients with or without DKD from a single center for a cross‐sectional study. Serum metabolic profiling was performed with MALDI‐TOF‐MS using a vertical silicon nanowire array. Differential metabolites between DKD and diabetes patients were selected, and their relevance to the clinical parameters of DKD was analyzed. We applied machine learning methods to the differential metabolite panels to distinguish DKD patients from diabetes patients. Twenty‐four differential serum metabolites between DKD patients and diabetes patients were identified, which were mainly enriched in butyrate metabolism, TCA cycle, and alanine, aspartate, and glutamate metabolism. Among the metabolites, l‐kynurenine was positively correlated with urinary microalbumin, urinary microalbumin/creatinine ratio (UACR), creatinine, and urea nitrogen content. l‐Serine, pimelic acid, 5‐methylfuran‐2‐carboxylic acid, 4‐methylbenzaldehyde, and dihydrouracil were associated with the estimated glomerular filtration rate (eGFR). The panel of differential metabolites could be used to distinguish between DKD and diabetes patients with an AUC value reaching 0.9899–0.9949. Among the differential metabolites, l‐kynurenine was related to the progression of DKD. The differential metabolites exhibited excellent performance at distinguishing between DKD and diabetes. This study provides a novel direction for metabolomics‐based clinical detection of DKD.

## Graphical abstract

Serum metabolic profiling of type II diabetes mellitus (T2DM) patients with or without diabetic kidney disease (DKD) were obtained with vertical silicon nanowires array‐assisted LDI‐TOF‐MS. Twenty‐four differential serum metabolites between DKD and T2DM were identified, mainly enriched in butyrate metabolism and the TCA cycle. Moreover, l‐kynurenine was related to the progression of DKD. The differential metabolites had excellent performance in distinguishing between DKD and T2DM.

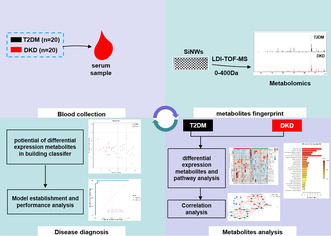

**Abbreviations**
CKD: chronic kidney disease
DKD: diabetic kidney disease
LASSO: least absolute shrinkage or selection operator
LDI‐TOF‐MS: laser desorption/ionization time‐of‐flight mass spectrometry
MALDI‐TOF‐MS: matrix‐assisted laser desorption/ionization time‐of‐flight mass spectrometry
NDKD: nondiabetic kidney disease
OPLS‐DA: orthogonal partial least squares discriminant analysis
PCA: principal component analysis
SiNW: silicon nanowires
SVM: support vector machine
T2DM: type II diabetes mellitus
UACR: urinary microalbumin/creatinine ratio

Diabetic kidney disease (DKD) is the most common complication of diabetes and the main cause of end‐stage renal disease [1, 2]. At present, the treatment for DKD mainly depends on blood glucose and hypertension control [3]. Despite the great efforts in blood glucose and blood pressure control, many patients eventually develop DKD [4, 5]. Although the detection of urinary protein content is the most common screening method for nephropathy in diabetes patients, the renal injury may have lasted for a long time before the significant clinical change of urinary albumin content. In addition, it is relatively difficult for the reverse treatment after the occurrence of proteinuria. Therefore, how to find DKD earlier is an important factor for early intervention and even reversing the progress of DKD.

Many studies have pointed out that, compared with patients with diabetes, the urine metabolic profiling in patients with DKD changes [6]. Abnormal metabolites in urine, such as 3‐hydroxy isovalerate, aconitic acid, citric acid, 2‐ethyl 3‐OH propionate, and 3‐hydroxy isobutyrate are also abnormal in the serum of patients with DKD [6]. In addition, the study of serum metabolomics revealed that the serum metabolic profiling was different between patients with DKD and patients with diabetes [7]. Serum metabolites, including hexadecanoic acid (C16:0), linolelaidic acid (C18:2N6T), linoleic acid (C18:2N6C), are potential markers to identify early DKD [7].

Matrix‐assisted laser desorption/ionization time‐of‐flight mass spectrometry (MALDI‐TOF‐MS) is widely used in clinical diagnosis because of its high‐throughput [8, 9]. Previous studies have found that a series of metabolites, including sugars and amino acids, have been found significantly changed in the kidney tissue of the rat model of DKD induced by high‐fat feeding and STZ through air‐flow‐assisted destruction electrospray ionization and matrix‐assisted laser destruction integrated mass spectroscopy imaging [10]. However, serum metabolic profiling in patients with DKD and diabetes using MALDI‐TOF‐MS has not been definitively reported. In addition, MALDI‐TOF‐MS is limited in the detection of metabolic profiling because of the background interference caused by the matrix. Meanwhile, silicon nanowires (SiNW) array chip can be used for MALDI‐TOF‐MS without adding matrix [11, 12], which makes it possible in high‐throughput detection of metabolic profiling in the clinic. Whether SiNW‐assisted laser desorption/ionization (LDI)‐TOF‐MS can be used to analyze differences in serum metabolic profiling between patients with DKD and those with diabetes mellitus to construct discriminative models remains to be investigated.

In this study we used the SiNW‐assisted LDI‐TOF‐MS technology to analyze the serum metabolic profiling of patients with DKD and diabetes, and analyzed the correlation between differential metabolites as related indicators for DKD progression. In addition, the performance of serum differential metabolite panel combined with machine learning in distinguishing DKD patients from diabetes patients was studied.

## Materials and methods

### Sample collection

This study was a cross‐sectional study. A total of 20 patients with diabetes and 20 patients with DKD were included in this study, all from the Department of Nephrology, Zhejiang Provincial People's Hospital. The protocol for this research project was approved by the Ethics Committee of Zhejiang Provincial People's Hospital (Approval No. 2021KY064) and conforms to the provisions of the Declaration of Helsinki. All oral informed consent was obtained from the subjects. Patients with Type II diabetes mellitus (T2DM) were recruited. Inclusion criteria: all patients were confirmed to be diagnosed with diabetes on the basis of the American Diabetes Association criteria with fasting plasma glucose ≥ 7.0 mmol·L^−1^, or hemoglobin A1c ≥ 6.5% or oral glucose tolerance test 2 h post‐load plasma glucose ≥ 11.1 mmol·L^−1^ or self‐reported medical history [13]. T1DM was excluded in this study, who had abnormal secretion of insulin, as manifested by low or undetectable levels of plasma C‐peptide, and abnormal expression of autoimmune markers, including islet cell autoantibodies and autoantibodies to GAD (GAD65), insulin, the tyrosine phosphatases IA‐2 and IA‐2b, and zinc transporter 8 [13]. The inclusion criteria for DKD were T2DM patients with proteinuria (urine albumin‐to‐creatinine ratio, namely, UACR ≥ 30 mg·g^−1^) or renal failure (estimated glomerular filtration rate [eGFR] < 60 mL·min^−1^·1.73 m^−2^) for at least 3 months [14, 15, 16]. 30 mg·g^−1^ ≤ UACR < 300 mg·g^−1^ is considered to be mcroalbuminuria, while UACR ≥ 300 mg·g^−1^ is considered as macroalbuminuria [17]. T2DM patients with the existence of chronic kidney disease (CKD) before DM was diagnosed were excluded. Diabetic patients without proteinuria (UACR < 30 mg·g^−1^) and with normal renal function (eGFR ≥ 60 mL·min^−1^·1.73 m^−2^) were used as controls and were included in the study. All patients were from the Department of Nephrology, Zhejiang Provincial People's Hospital. We began continuous enrollment of T2DM patients on December 19, 2021, with or without DKD. When the number of patients in one group reached 20, the recruitment of this group of patients was discontinued. Finally, 20 patients with DKD and 20 patients with T2DM were obtained. Blood samples were collected the next morning after fasting. Blood samples were centrifuged to prepare serum samples, which were then frozen at −80 °C.

### Serum sample pretreatment

Methanol solution was added to the serum and the mixture was shaken and centrifuged at 2200 ***g***. Then MTBE solution was added and the mixture was shaken and centrifuged at 2200 ***g***. The water was added and the mixture was centrifuged, so that the mixture solution was divided into upper and lower layers. The upper and lower solutions were sucked out simultaneously, mixed, and blow‐dried with nitrogen. The mixed layer sample was taken and 50% methanol solution was added to resuspend the mixture, which was then frozen at −80 °C.

### LDI‐TOF‐MS measurement

Serum metabolite extract was shaken for 5 min and spotted on the vertical silicon nanowires (SINWs) array (a disposable matrix free mass spectrometry chip) [11] (Lip‐Si Array™; Hangzhou Huijian Technology Co., Ltd, Hangzhou, China), and the chip was tested on the mass spectrometer (Bruker Daltonics Inc., Billerica, MA, USA). The instrument is equipped with a Smartbeam™ II solid state laser (Brussels, Belgium; pulse energy < 500 μJ, pulse width = 3 ns). The diameter of the laser spot was set to 80–100 μm. The relative laser energy was set at 55–63% of the maximum energy. The ions produced by 100 ns pulsed ion extraction were subjected to electric fields of 19.18 kV (ion source 1) and 16.92 kV (ion source 2) and the sample was analyzed by reflection mode. The cumulative number of laser shots per hole was 1250 shots per hole.

### Statistical analysis

The missing value was moved according to the 80% rule, wherein a metabolite was considered detectable when it was detected across at least 4/5 samples in one group [18]. The normalization algorithm used here were normalization to MS “total useful signal” [19]. To remove potential interbatch variations, the correlation coefficients between serum metabolic profiles of three patients with DKD and three patients with DM were analyzed.

Principal component analysis (PCA) and orthogonal partial least‐squares discriminant analysis (OPLS‐DA) were performed on the R language metaboanalystr 3.1.0 package (Vienna, Austria). Based on the nonparametric Wilcoxon test and VIP value, differential metabolites were selected (*P* < 0.05 and VIP > 1) and identified by the HMDB database. *P* < 0.05 means that the difference was statistically significant. The correlation analysis was performed by using the corr.test function of R language and the hetcor function of the polycor package. The correlation network was drawn by cytoscape software [20]. Support Vector Machine (SVM), the least absolute shrinkage or selection operator (LASSO) regression, was used to establish a discriminant model conducted in R language package. ROC analysis was performed by the ROC function of R language.

## Results

### Study population

Twenty patients were DKD patients and 20 patients were T2DM patients without DKD. All participants in each sample group participated in a diabetes treatment intervention. All DKD patients had abnormal UACR (≥30 mg·g^−1^), and 18 patients with microalbuminuria (30 mg·g^−1^ ≤ UACR < 300 mg·g^−1^), two patients with macroalbuminuria (UACR > 300 mg·g^−1^). The mean UACR of all patients with DKD was 129.6 mg·g^−1^. Six patients with DKD were accompanied with renal failure, namely eGFR < 60 mL·min^−1^·1.73 m^−2^, the mean eGFR reached 76.9 mL·min^−1^·1.73 m^−2^ (Table 1). Data showed that there was a significant difference in age between the diabetes group and the DKD group, and the latter was significantly older than the former (*P* < 0.01); there was no significant difference in sex ratio and age of onset of diabetes between the two groups (Table 1). However, the duration of diabetes in patients with DKD was significantly longer than that in patients with diabetes; the average of the former was 3.8 years and the latter was 10.8 years (Table 1). In terms of the incidence of diabetes complications, the incidence of retinopathy and hypertension in patients with DKD was significantly higher than that in patients with diabetes, and there was a statistical difference (both *P* < 0.01, Table 1). In addition, in terms of total protein, albumin, and blood glucose, there was no significant difference in content or value between patients with diabetes and patients with DKD (*P* > 0.05, Table 1). The contents of creatinine and urea nitrogen in patients with DKD were significantly higher than those in the diabetes group (*P* < 0.001, *P* < 0.05, respectively), while the eGFR value in the diabetes group was significantly higher than that in patients with DKD (*P* < 0.001) (Table 1). It showed that the renal function of patients with DKD was significantly impaired compared with that of patients with diabetes. Only one person in the diabetes group received antihypertensive treatment, and none received ACE inhibitor treatment; 16 people in the DKD group received ACE inhibitors or antihypertensive therapy. Differences in the number of patients receiving ACE inhibitors or antihypertensive therapy between groups were associated with a greater number of hypertensive patients in the DKD group.

**Table 1:** Clinical characteristics of patients with diabetes and DKD. The value is expressed as mean ± SD. Differences of the mean value and classification between groups evaluated with Student's *t*‐test and Fisher's exact test, respectively. The *P* values in bold are statistically significant (*P* < 0.05). ALB, albumin; BUN, urea nitrogen; CRE, creatinine; mALB, urinary microalbumin; TP, total protein.

Clinical features	Diabetes (*n* = 20)	DKD (*n* = 20)	*P* value
Age (years)	48.4 ± 11.5	59.7 ± 12.8	0.0055
Sex (male/female)	13/7	14/6	1.0000
Blood glucose (mmol·L^−1^)	6.2 ± 1.7	6.1 ± 2.2	0.8777
Diabetes treatment intervention (yes/no)	20/0	20/0	1.0000
Other metabolic states			
Hyperuricemia (yes/no)	3/17	4/16	1.0000
Hyperlipemia (yes/no)	8/12	9/11	1.0000
BMI (kg/m^2^)	24.5 ± 3.6	24.6 ± 2.5	0.9158
Smoking (yes/no)	8/12	5/15	0.5006
Smoker (former/current)	4/4	3/2	1.0000
Age at diabetes onset (years)	44.6 ± 11.0	49.4 ± 13.4	0.2276
Diabetes duration (years)	3.8 ± 5.6	10.8 ± 8.1	0.0029
Retinopathy (yes/no)	0/20	8/12	0.0033
Hypertension (yes/no)	2/18	12/8	0.0022
ACE inhibitors or antihypertensive therapy (yes/no)	1/19	16/4	< 0.0001
mALB (positive/negative)	0/20	20/0	< 0.0001
mALB (mg·L^−1^)	NA	763.2 ± 1240.1	
UACR (positive/negative)	0/20	20/0	< 0.0001
UACR (mg·g^−1^)	NA	129.6 ± 279.7	
Microalbuminuria (yes)	0	18	
Macroalbuminuria (yes)	0	2	
TP (g·L^−1^)	66.3 ± 6.1	66.0 ± 7.1	0.8827
ALB (g·L^−1^)	38.3 ± 3.0	36.5 ± 5.3	0.1901
BUN (mmol·L^−1^)	4.3 ± 1.2	8.9 ± 5.6	0.0009
CRE (μmol·L^−1^)	69.3 ± 11.5	120.7 ± 90.7	0.0163
eGFR (mL·min^−1^·1.73 m^−2^)	113.0 ± 21.6	76.9 ± 35.0	0.0004

### Analysis of serum metabolic profiling in patients with diabetes and DKD

**Fig. 1:**
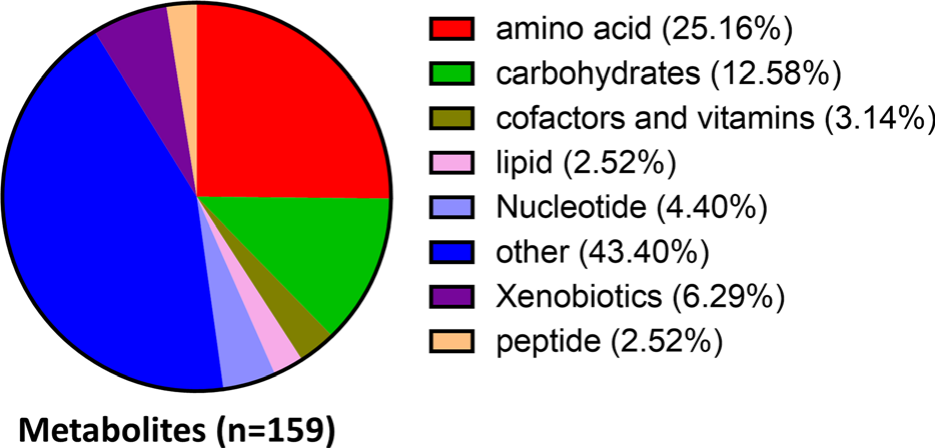
The numbers and proportions of metabolites in our study.

Using SiNW‐assisted LDI‐TOF‐MS, we found 420 peaks of metabolites in more than 80% of the serum samples from DM and DKD patients. A total of 159 metabolites were identified by the HMDB database, and some of them belong to amino acid and carbohydrates (Fig. 1). To test the interbatch variability of metabolic profiles, serum metabolic profiling from three patients with diabetes and three patients with DKD in triplicate was collected, and correlation coefficient analysis was conducted. It was found that the correlation coefficient between metabolic profiling obtained from the same patient among repeated tests was above 0.8, while the correlation coefficient between metabolic profiling of samples from different patients was mostly lower than 0.8 (Fig. S1), and this result indicated that the procedure for serum metabolic profiling was credible and stable.

**Fig. 2:**
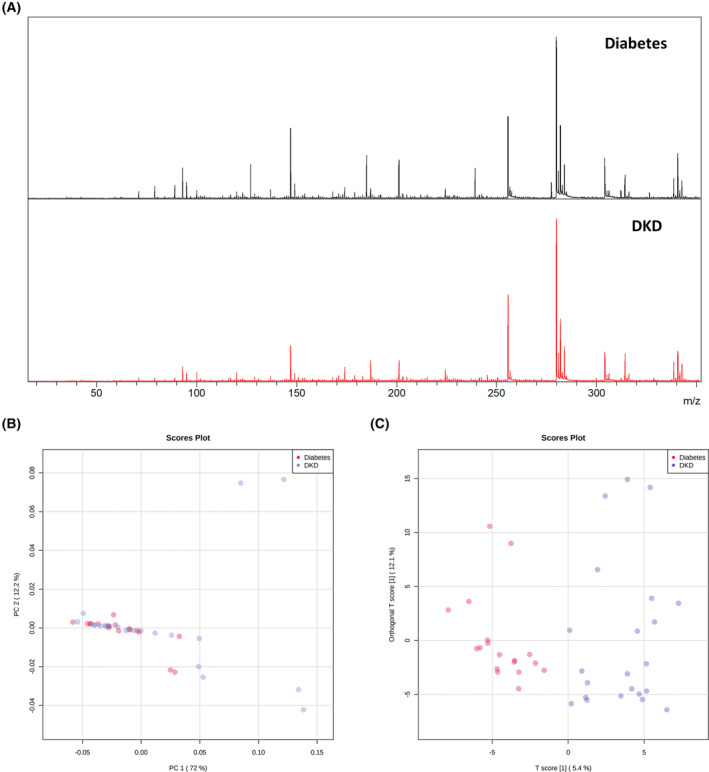
PCA and OPLS‐DA of serum metabolic profiling from patients with diabetes and DKD. (A) Mass spectra of serum metabolites profiling in patients with diabetes and patients with DKD; (B) PCA score plot (all peaks); (C) OPLS‐DA score plot (all peaks).

It could be seen that serum metabolic profiling of patients with diabetes and patients with DKD were different (Fig. 2A). The correlation coefficient between metabolic profiling of different samples in the DKD group was higher than that of metabolic profiling of different samples in diabetes patients and DKD patients (Fig. S1), which indicated that there were differences in serum metabolic profiling between diabetes patients and DKD patients. Through unsupervised learning PCA of serum metabolic profiling of patients with diabetes and patients with DKD, it was found that the discrimination between the two groups was not very obvious, and the best separation between the groups was again observed in PC1 and PC2 (12.2% and 72% of the observed variance, respectively) (Fig. 2B). However, remarkably, an almost complete separation of the DKD group from the diabetes group could be observed in OPLS‐DA (Fig. 2C). This indicated that the serum metabolic profiling between diabetes patients and DKD patients was different.

### Analysis of differential metabolites and metabolic pathways in serum from patients with DKD

**Fig. 3:**
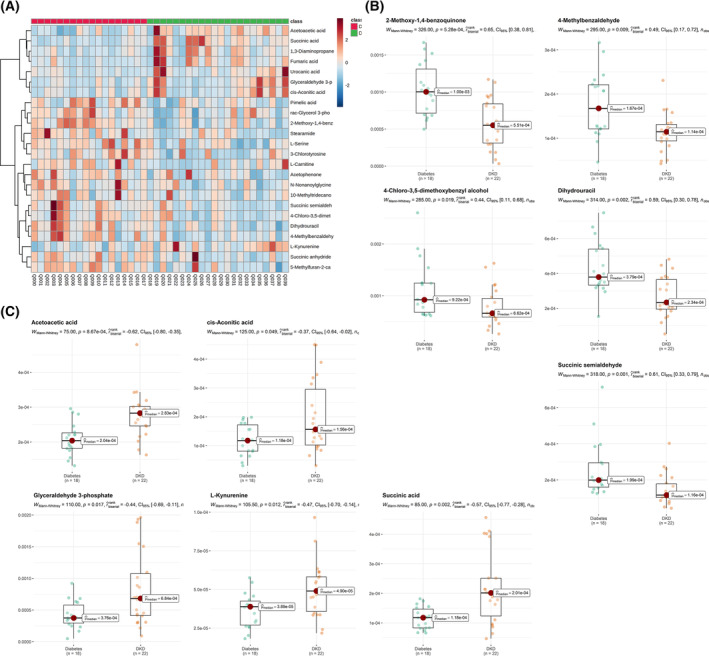
Heatmap and relative content box diagram of 24 differential metabolites in serum from patients with diabetes and patients with DKD. (A) Cluster heat map of differential metabolites; Distance measure was the Euclidean. For clustering heatmaps, the data were ploted by Pheatmap package in R(4.1.3), according to Ward's method. (B) Downregulated top5 metabolites with highest VIP value in serum of patients with DKD. (C) Upregulated top5 metabolites with the highest VIP value in serum of patients with DKD.

We found that among the metabolites mentioned above, 24 metabolites were significantly changed in the serum of patients with DKD compared with those patients with diabetes, which met *P* < 0.05 and VIP value > 1 (Table 2 and Fig. 3A). The information about those 24 differential metabolites is summarized in Table 3. Compared with patients with diabetes, there were 16 downregulated metabolites in the serum of patients with DKD, namely, dihydrouracil, acetophenone, l‐carnitine, l‐serine, *N*‐nonanoylglycine, l‐stearamide, 3‐chlorotyrosine, 4‐chloro‐3,5‐dimethoxybenzyl alcohol, 4‐methylbenzaldehyde, 5‐methylfuran‐2‐carboxylic acid, 2‐methoxy‐1,4‐benzoquinone, 10‐methyltridecanoic acid, succinic semialdehyde, succinic anhydride, rac‐glycerol 3‐phosphate, and pimelic acid (Tables 2 and 3 and Fig. 3B). While, the content of eight metabolites in the serum of patients with DKD was higher than those patients with diabetes, which are acetoacetic acid, 1,3‐diaminopropane, fumaric acid, succinic acid, cis‐aconitic acid, urocanic acid, glyceraldehyde 3‐phosphate, and l‐kynurenine (Tables 2 and 3 and Fig. 3C). Among them, the 10 metabolites with the largest VIP value were dihydrouracil (VIP = 2.658), acetoacetic acid (VIP = 2.608), 2‐methoxy‐1,4‐benzoquinone (VIP = 2.572), succinic semialdehyde (VIP = 2.466), 4‐methylbenzaldehyde (VIP = 2.308), 4‐chloro‐3,5‐dimethoxybenzyl alcohol (VIP = 2.255), succinic acid (VIP = 2.108), and glycyraldehyde 3‐phosphate (VIP = 2.085), *N*‐nonanoylglycine (VIP = 2.030), stearamide (VIP = 2.023) (Table 3).

**Table 2:** Identified differential metabolites between diabetes patients and DKD patients. *P*‐value is calculated according to the Wilcoxon test.

Metabolite	*P*‐value	VIP [t]	VIP [ortho‐t]	Formula	HMDB.ID
1,3‐Diaminopropane	0.047914	1.58714133	1.358163166	C3H10N2	HMDB0000002
10‐Methyltridecanoic acid	0.039134	1.778738019	1.149951164	C14H28O2	HMDB0061781
2‐Methoxy‐1,4‐benzoquinone	0.00030218	2.571975689	1.099891672	C7H6O3	HMDB0032576
3‐Chlorotyrosine	0.010767	1.000833502	0.669933508	C9H10ClNO	HMDB0001885
4‐Chloro‐3,5‐dimethoxybenzyl alcohol	0.017477	2.254599151	0.972580032	C9H11ClO3	HMDB0040933
4‐Methylbenzaldehyde	0.0076514	2.30802472	0.3439665	C8H8O	HMDB0029638
5‐Methylfuran‐2‐carboxylic acid	0.0020387	2.006495112	0.689942502	C6H6O3	HMDB0059735
Acetoacetic acid	0.00055051	2.608116022	0.909854511	C4H6O3	HMDB0000060
Acetophenone	0.029538	1.942685335	0.527691639	C8H8O	HMDB0033910
cis‐Aconitic acid	0.047914	1.964942812	0.770927836	C6H6O6	HMDB0000072
Dihydrouracil	0.0012068	2.658011983	0.298411048	C4H6N2O2	HMDB0000076
Fumaric acid	0.023711	1.579971342	1.375757239	C4H4O4	HMDB0000134
Glyceraldehyde 3‐phosphate	0.016158	2.085106078	0.78236088	C3H7O6P	HMDB0001112
l‐Carnitine	0.0083455	1.408128624	0.244362146	C7H15NO3	HMDB0000062
l‐Kynurenine	0.011699	1.814767932	0.296423242	C10H12N2O3	HMDB0000684
l‐Serine	0.0044509	1.811871453	1.026615502	C3H7NO3	HMDB0000187
*N*‐Nonanoylglycine	0.011699	2.029623335	1.078564462	C11H21NO3	HMDB0013279
Pimelic acid	0.0076514	1.800121615	1.069675979	C7H12O4	HMDB0000857
rac‐Glycerol 3‐phosphoate	0.010767	1.455720802	1.287920126	C3H9O6P	HMDB0000126
Stearamide	0.0048838	2.023308001	0.549736044	C18H37NO	HMDB0034146
Succinic acid	0.0016588	2.107715391	0.905104167	C4H6O4	HMDB0000254
Succinic anhydride	0.021999	1.475214919	0.540712293	C4H4O3	HMDB0032523
Succinic semialdehyde	0.0007765	2.465880197	0.631098969	C4H6O3	HMDB0001259
Urocanic acid	0.044824	1.405040791	1.236454863	C6H6N2O2	HMDB0000301

**Table 3:** Information summary for the 24 differential metabolites between diabetes patients and DKD patients.

Category	Metabolite	DKD vs. T2DM	Metabolic pathway
Unknown	10‐Methyltridecanoic acid	Downregulation	Unknown
Unknown	2‐Methoxy‐1,4‐benzoquinone	Downregulation	Unknown
Unknown	3‐Chlorotyrosine	Downregulation	Unknown
Unknown	4‐Chloro‐3,5‐dimethoxybenzyl alcohol	Downregulation	Unknown
Xenobiotics biodegradation and metabolism	4‐Methylbenzaldehyde	Downregulation	Xylene degradation
Unknown	5‐Methylfuran‐2‐carboxylic acid	Downregulation	Unknown
Xenobiotics biodegradation and metabolism	Acetophenone	Downregulation	Ethylbenzene degradation
Nucleotide metabolism	Dihydrouracil	Downregulation	Pyrimidine metabolism; beta‐alanine metabolism; pantothenate and CoA biosynthesis
Unknown	l‐Carnitine	Downregulation	Unknown
Amino acid metabolism	l‐Serine	Downregulation	Glycine, serine and threonine metabolism; cysteine and methionine metabolism; d‐amino acid metabolism; glycerophospholipid metabolism; sphingolipid metabolism; glyoxylate and dicarboxylate metabolism
Unknown	*N*‐Nonanoylglycine	Downregulation	Unknown
Metabolism of cofactors and vitamins	Pimelic acid	Downregulation	Biotin metabolism; biosynthesis of cofactors
Unknown	rac‐Glycerol 3‐phosphoate	Downregulation	Unknown
Unknown	Stearamide	Downregulation	Unknown
Carbohydrate metabolism	Succinic anhydride	Downregulation	Citrate cycle (TCA cycle); oxidative phosphorylation; alanine, aspartate and glutamate metabolism; lysine degradation; tyrosine metabolism; phenylalanine metabolism; pyruvate metabolism; glyoxylate and dicarboxylate metabolism; propanoate metabolism; butanoate metabolism; nicotinate and nicotinamide metabolism; sulfur metabolism
Amino acid metabolism	Succinic semialdehyde	Downregulation	Alanine, aspartate and glutamate metabolism; tyrosine metabolism; butanoate metabolism; vitamin B6 metabolism; nicotinate and nicotinamide metabolism
Amino acid metabolism	Urocanic acid	Upregulation	Histidine metabolism
Amino acid metabolism	Acetoacetic acid	Upregulation	Valine, leucine and isoleucine degradation; lysine degradation; tyrosine metabolism; styrene degradation; butanoate metabolism
Carbohydrate metabolism	cis‐Aconitic acid	Upregulation	Citrate cycle (TCA cycle); glyoxylate and dicarboxylate metabolism
Carbohydrate metabolism	Fumaric acid	Upregulation	Citrate cycle (TCA cycle); oxidative phosphorylation; arginine biosynthesis; alanine, aspartate and glutamate metabolism; tyrosine metabolism; phenylalanine metabolism; pyruvate metabolism; styrene degradation; butanoate metabolism; nicotinate and nicotinamide metabolism
Amino acid metabolism	l‐Kynurenine	Upregulation	Tryptophan metabolism
Carbohydrate metabolism	Glyceraldehyde 3‐phosphate	Upregulation	Glycolysis / Gluconeogenesis; pentose phosphate pathway; fructose and mannose metabolism; galactose metabolism; clavulanic acid biosynthesis; inositol phosphate metabolism; methane metabolism; thiamine metabolism; vitamin B6 metabolism
Carbohydrate metabolism	Succinic acid	Upregulation	Citrate cycle (TCA cycle); oxidative phosphorylation; alanine, aspartate and glutamate metabolism; lysine degradation; tyrosine metabolism; phenylalanine metabolism; pyruvate metabolism; glyoxylate and dicarboxylate metabolism; propanoate metabolism; butanoate metabolism; nicotinate and nicotinamide metabolism; sulfur metabolism
Amino acid metabolism	1,3‐Diaminopropane	Upregulation	Glycine, serine and threonine metabolism; arginine and proline metabolism; beta‐alanine metabolism

**Fig. 4:**
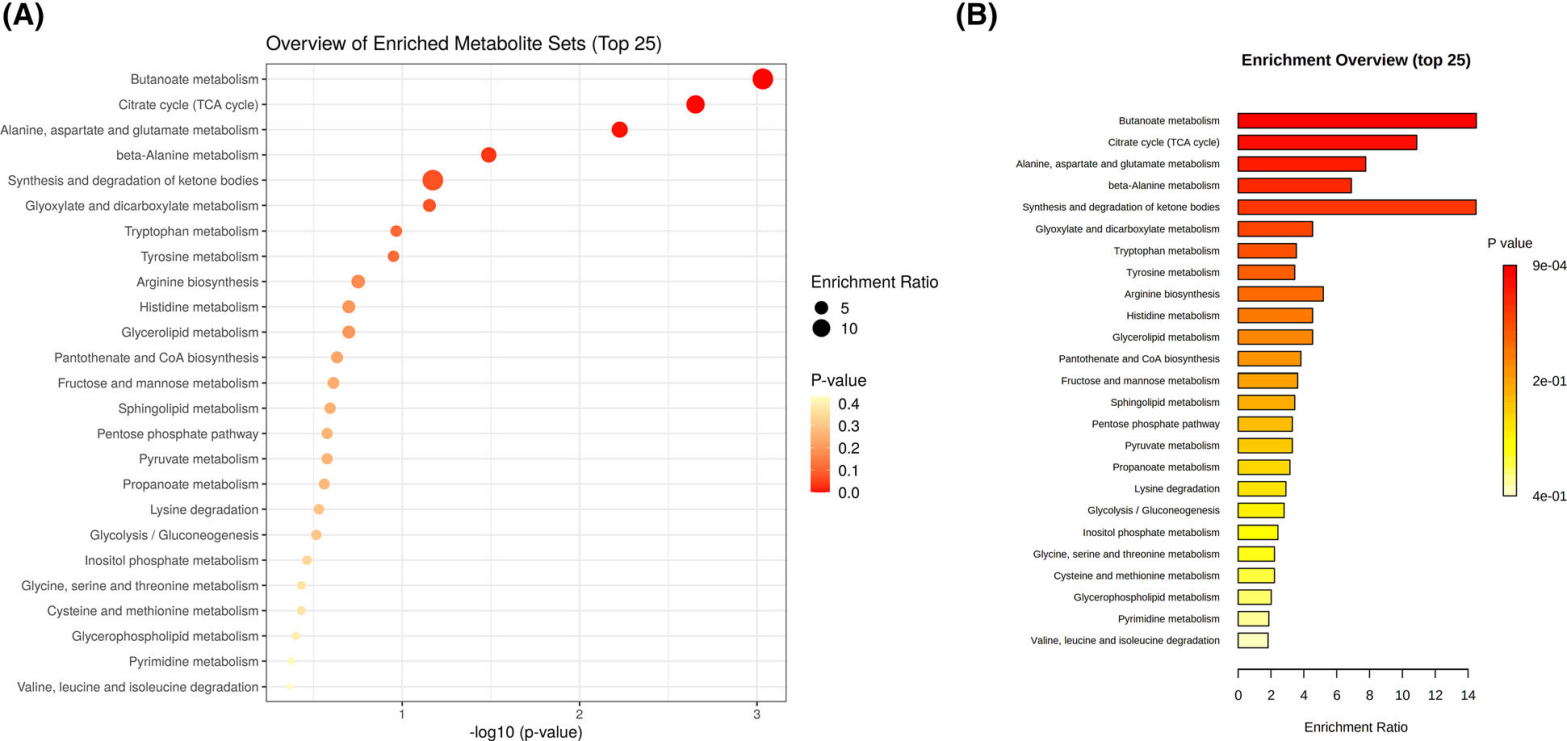
Enrichment analysis of metabolic pathways in serum from patients with diabetes and patients with DKD. (A) Bubble diagram of Top 25 metabolic pathway enrichment analysis; the horizontal axis represents the significance, and the point size represents the enrichment rate of metabolites. (B) Bar graph of Top 25 metabolic pathway enrichment analysis. Color represents *P*‐value.

The enrichment analysis of metabolic pathways based on the above 24 differential metabolites showed that, compared with diabetes patients, the serum metabolic pathways of patients with DKD were disordered, including butyrate metabolism, TCA cycle, alanine, aspartate and glutamate metabolism, mainly involved in carbohydrate metabolism and amino acid metabolism (Table 3 and Fig. 4).

### Correlation analysis between differential metabolites and demographic and clinical characteristics

**Fig. 5:**
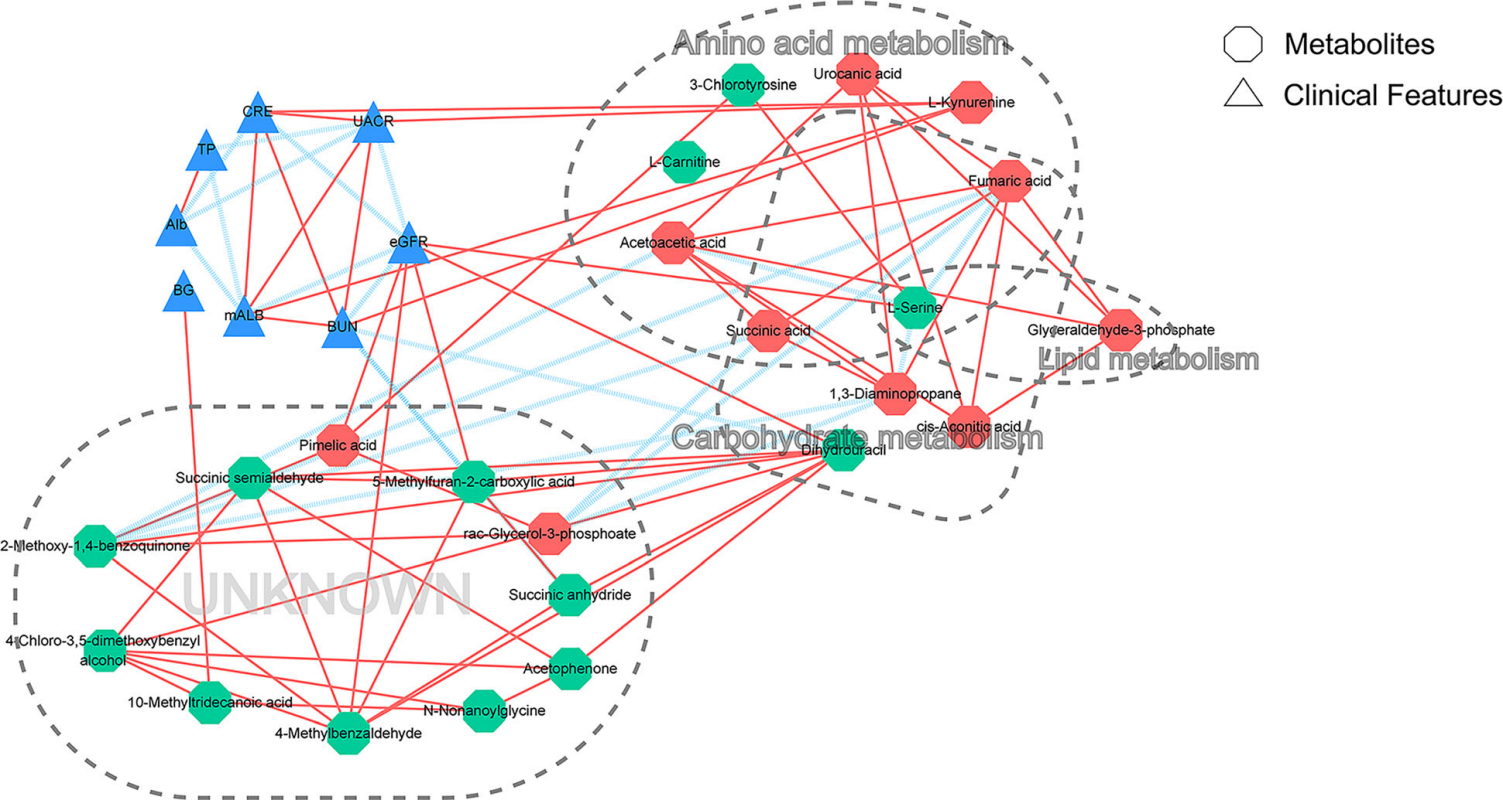
Correlation network of differential metabolites and biochemical indexes. The substances or nodes with significant correlation were screened out (*P* < 0.05 after FDR correction), and the correlation network was drawn by cytoscape software [20]. Circles, differential metabolites; triangle, clinical features, including blood glucose (BG), total protein (TP), albumin (ALB), urinary microalbumin (mALB), UACR, urea nitrogen (BUN), creatinine (CRE), and eGFR. Red solid line, positive correlation; blue dotted line, negative correlation. Red indicates the upregulated differential metabolites in patients with DKD; green indicates the downregulated differential metabolites in patients with DKD.

Through the correlation analysis of differential metabolites with population characteristics and clinical characteristics, it was found that the differential metabolites significantly related to age were 5‐methylfuran‐2‐carboxylic acid, 4‐methylbenzaldehyde, and succinic anhydride, all of which were negatively correlated with ages; the differential metabolites with significant correlation with sex were succinic acid, urocanic acid, fumaric acid, and 4‐methylbenzaldehyde (Fig. S2). Moreover, the only metabolite significantly correlated with blood glucose was 10‐methyltridecanoic acid (Fig. S2).

l‐Kynurenine was a metabolite significantly related to urinary mALB, UACR, creatinine, and urea nitrogen content (Fig. S2 and Fig. 5). While, 5‐methylfuran‐2‐carboxylic acid and dihydrouracil were the metabolites that had a significant negative correlation with urea nitrogen content; the metabolites correlated with eGFR were l‐serine, pimelic acid, 5‐methylfuran‐2‐carboxylic acid, 4‐methylbenzaldehyde, and dihydrouracil, all of which were positively correlated with eGFR (*P* < 0.05, Fig. S2 and Fig. 5).

Correlation analysis of differential metabolites showed that, except for l‐carnitine and stearamide, there was a significant correlation among the remaining 21 differential metabolites (*P* < 0.05, Fig. S3 and Fig. 5).

There were positive correlations among urinary mALB, UACR, creatinine, and urea nitrogen. Urinary mALB, UACR, creatinine, and urea nitrogen were all negatively correlated with eGFR; there was no correlation between blood glucose and biochemical indexes related to diabetes DKD. Total protein and albumin were negatively correlated with UACR and urinary mALB (Fig. 5). Differential metabolites with significant correlation were involved in amino acid metabolism, carbohydrate metabolism, and lipid metabolism (Fig. 5). l‐Kynurenine was the serum differential metabolite that had the most correlation with biochemical indexes related to DKD; among the biochemical indicators related to DKD, the number of metabolites was most significantly related to eGFR, followed by urea nitrogen, and the remaining indicators were following along, which were significantly related to l‐kynurenine (Fig. 5).

### Performance analysis of differential metabolites panel in differentiating patients with diabetes and DKD

**Fig. 6:**
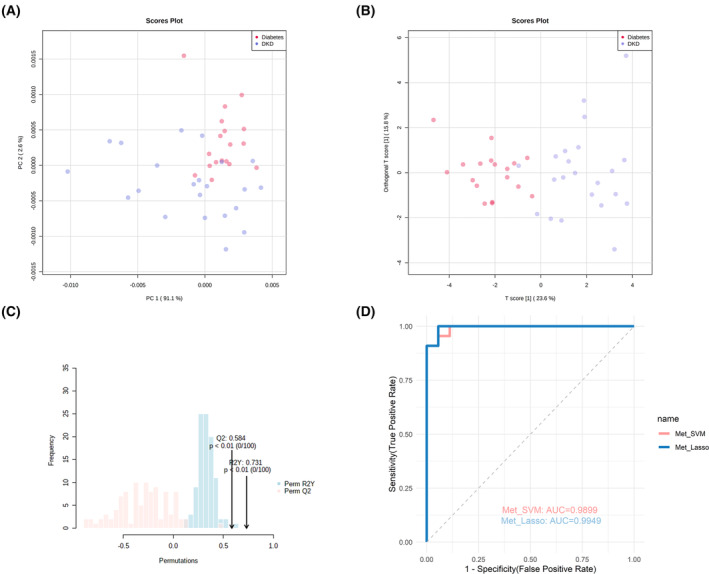
PCA and OPLS‐DA and its permutation tests of differential metabolites in serum from patients with diabetes and patients with DKD, and ROC curves of different classifiers. (A) PCA score plot; (B) OPLS‐DA score plot; (C) Validation plot obtained from 100 times of permutation tests; (D) ROC curve of classifiers based on 24 differential metabolites or clinical features in distinguishing patients with DKD from patients with diabetes. Met_SVM, SVM model was built using a metabolite panel consisting of 24 differential metabolites; Met_LASSO, LASSO was applied to the build model.

First, based on 24 differential metabolites, unsupervised PCA analysis and supervised OPLS‐DA analysis was performed. The results showed that the two groups were significantly distinguished (Fig. 6A,B). In the OPLS‐DA model, *Q* ^2^ reached 0.579 and *R* ^2^ *Y* reached 0.73, both higher than 0.5, indicating that the 24 differential metabolites had good predictive ability in distinguishing diabetes patients and DKD patients (Fig. 6B). In addition, the validity of the OPLS‐DA model was confirmed using permutation test (Fig. 6C), indicating that the model was not overfitting.

Subsequently, the performance of 24 differential metabolites in distinguishing patients with diabetes and patients with DKD were analyzed. It was found that the AUC values of the remaining 20 metabolites were higher than 0.7, except that the AUC values of the four metabolites 10‐methylridecanoic acid, urocanic acid, cis‐aconitic acid, and 1,3‐diaminoprotein were 0.68–0.70. The metabolite with the highest AUC value was 2‐methoxy‐1,4‐benzoquinone (AUC = 0.8232) (Table 4). It was suggested that these 24 differential metabolites maybe have good performance in distinguishing between patients with diabetes and patients with DKD.

**Table 4:** AUC values of differential metabolites in differentiating between patients with diabetes and patients with DKD.

Metabolite	AUC
2‐Methoxy‐1,4‐benzoquinone	0.823232
Acetoacetic acid	0.810606
Succinic semialdehyde	0.803030
Dihydrouracil	0.792929
Succinic acid	0.785354
5‐Methylfuran‐2‐carboxylic acid	0.780303
l‐Serine	0.760101
Stearamide	0.758838
4‐Methylbenzaldehyde	0.744949
Pimelic acid	0.744949
l‐Carnitine	0.742424
rac‐Glycerol 3‐phosphoate	0.734848
3‐Chlorotyrosine	0.734848
*N*‐Nonanoylglycine	0.732323
l‐Kynurenine	0.732323
Glyceraldehyde 3‐phosphate	0.722222
4‐Chloro‐3,5‐dimethoxybenzyl alcohol	0.719697
Succinic anhydride	0.712121
Fumaric acid	0.709596
Acetophenone	0.702020
10‐Methyltridecanoic acid	0.691919
Urocanic acid	0.686869
cis‐Aconitic acid	0.684343
1,3‐Diaminopropane	0.684343

Based on the 24 differential metabolites, a different machine‐learning method SVM or LASSO regression was applied to build a classifier to distinguish patients with DKD and patients with diabetes, respectively. The SVM model consisted of all 24 metabolites, while the LASSO model consisted of Dihydrouracil, 4‐Methylbenzaldehyde, 5‐Methylfuran‐2‐carboxylic acid, Pimelic acid, Glyceraldehyde 3‐phosphate, 2‐Methoxy‐1,4‐benzoquinone, *N*‐Nonanoylglycine, 10‐Methyltridecanoic acid, Fumaric acid, Succinic acid, l‐Carnitine, and 3‐Chlorotyrosine, l‐Kynurenine (Table 5). It was found that the AUC value of the SVM model and the LASSO model reached 0.9899 and 0.9949, when identifying patients with DKD and patients with diabetes, which was higher than any single metabolite (Fig. 6D, Tables 4 and 5). The sensitivity and specificity of the metabolite panel were 90.91% and 100% for the SVM model, 95.45% and 94.44% for the LASSO model, respectively (Table 5). It showed that the metabolite panel had excellent performance in identifying patients with DKD from patients with diabetes.

**Table 5:** Performance of a metabolite panel, urinary exosomal miR‐486‐5p, and clinical features in differentiating patients with diabetes and patients with DKD.

Classifier names	Features	AUC	Sensitivity	Specificity
Met_SVM	24 differential metabolites	0.9899	90.91%	100%
Met_LASSO	Dihydrouracil, 4‐Methylbenzaldehyde, 5‐Methylfuran‐2‐carboxylic acid, Pimelic acid, Glyceraldehyde 3‐phosphate, 2‐Methoxy‐1,4‐benzoquinone, *N*‐Nonanoylglycine, 10‐Methyltridecanoic acid, Fumaric acid, Succinic acid, l‐Carnitine, 3‐Chlorotyrosine, l‐Kynurenine	0.9949	95.45%	94.44%

## Discussion

Spectrometry methods, particularly MALDI‐TOF‐MS, enable high‐throughput extraction and measurement of metabolomic information, while tandem MS allows accurate identification of metabolites [12]. When MALDI‐TOF‐MS is conducted, it is necessary to add matrix for desorption and ionization of the substance to be measured. The added matrix, such as the conventional organic matrix (α‐cyano‐4‐hydroxycinnamic acid), showed strong interference in the low mass range, and the sample pretreatment time is prolonged due to matrix spraying [14]. The SiNW‐based LDI‐TOF‐MS could avoid matrix interference and improve detection accuracy [15]. In this study, we used SiNW‐based LDI‐TOF‐MS to detect the metabolic profiling. It was found that the serum metabolic profiling of DKD patients was different from that of diabetes patients, and 24 differential metabolites were identified. The contents of dihydrouracil in serum or urine of DKD model mice were abnormal [19]. The level of succinic acid, cis‐aconitic acid, and kynurenine in serum or urine of patients with DKD were changed [6, 21, 22]. In this study we found that the contents of dihydrouracil, succinic acid, cis‐aconitic acid, and l‐kynurenine in the serum of DKD patients were also abnormal, indicating that the SiNW‐assisted LDI‐TOF‐MS‐based platform was credible for the detection of metabolic profiling.

Metabolic pathway enrichment analysis showed that the differential metabolites of DKD patients were mainly involved in butyric acid metabolism, tricarboxylic acid cycle, alanine, aspartic acid and glutamic acid metabolism, β‐alanine metabolism, and ketone synthesis and degradation. Some studies have pointed out that urinary tricarboxylic acid cycle is abnormal in patients with early DKD [23]. In addition, amino acid metabolism in serum and plasma of patients with diabetes was abnormal, including leucine metabolite, isoleucine metabolite, and valine metabolite [6, 24]. It showed that the serum tricarboxylic acid cycle of DKD patients changed in this study, which was consistent with previous studies; however, alanine, aspartic acid and glutamic acid metabolism are the most variable metabolic pathways in amino acid metabolism, which might be caused by the heterogeneity of DKD patients. In addition, butyric acid metabolism has been rarely studied in previous reports on DKD, which may play an important regulatory role in the pathogenesis of DKD and needs to be further studied and confirmed.

Studies have found that serum metabolites, for example, c‐glycosyltryptophan and pseudouridine, are associated with decreased renal function [16, 25], and tryptophan and kynurenine are related to eGFR [11, 18, 26]. In this study we found that l‐serine, pimelic acid, 5‐methylfuran‐2‐carboxylic acid, 4‐methylbenzaldehyde, and dihydrouracil were positively correlated with eGFR in all patients, which might be predictors of renal disease progression in patients with DKD. In addition, l‐kynurenine was positively correlated with urinary mALB, UACR, creatinine, and urea nitrogen, but not with eGFR. In the process of DKD, proteinuria often appears before eGFR decreases. It was suggested that l‐kynurenine might be a metabolic biomarker for the early diagnosis and predictor of DKD, which needs to be further investigated. At present, the early diagnosis and screening of DKD and the monitoring of DKD progression have become clinical difficulties. Metabolomics may be one of the feasible omics to find markers for early diagnosis and screening of DKD and to monitor the progression of DKD.

Several studies have pointed out that a metabolite panel could distinguish patients with DKD and patients with diabetes. For example, a metabolite panel consisted of γ‐butyrobetaine, SDMA, azelaic acid, and two unknowns, was applied for the multiple logistic regression model and the AUC value for diagnosing DKD was 0.880–0.927 [24]. In this study we found that when 24 differential metabolites were applied to the SVM model, or LASSO model, the AUC value for identifying DKD patients from diabetes patients reached 0.9899, 0.9949, respectively. In addition, the sensitivity and specificity of the metabolite panel was 90.91% and 100%, 95.45% and 94.44%, respectively, suggesting that the SiNW‐assisted LDI‐TOF‐MS‐based platform for metabolic profiling had the potential for clinical application in the diagnosis of DKD.

In another study, we also found differences in eGFR between T2DM patients and DKD patients [27], indicating that eGFR alterations are widespread in DKD patients. In this study we analyzed the correlation between the content of various clinical indicators and found that eGFR was significantly negatively correlated with UACR, suggesting that the decrease of eGFR in DKD patients was mainly related to changes in proteinuria (Fig. S4). We also analyzed the correlation coefficients between metabolites and clinical parameters and found that l‐kynurenine was significantly correlated with UACR, suggesting that l‐kynurenine was closely related to DKD (Fig. S2).

Although renal needle biopsy is the gold standard for DKD diagnosis, DKD diagnosis is currently mainly achieved through long‐term monitoring (>3 months) of proteinuria and eGFR. However, the detection of proteinuria is prone to transient proteinuria due to its high variability [28]. eGFR tests are often based on creatinine, which is susceptible to metabolite interference, such as levamisole [29]. Therefore, the existing screening and diagnosis methods are not adequate, and long‐term monitoring is required, which leads to prolonged diagnosis time and a low diagnosis rate of DKD due to discontinuation of follow‐up. At the same time, there is a high proportion of nondiabetic kidney disease (NDKD) in T2DM patients with CKD [30], and its prognosis and treatment are significantly different from DKD [31, 32]. At present, kidney biopsy is the only feasible method for the differential diagnosis of DKD and NDKD. Therefore, there is a lack of noninvasive detection methods for the early diagnosis and screening of DKD. MALDI‐TOF‐MS has been widely used in clinical microbiological detection [8, 33]. Conventional matrix‐assisted LDI‐TOF‐MS detection technology produces background interference in the metabolite region, while the SiNW‐assisted LDI‐TOF‐MS platform can solve the technical difficulties. And in the differential metabolites panel we found, based on this technology platform, can effectively distinguish patients with T2DM and DKD. These findings suggested that SiNW‐assisted LDI‐TOF‐MS‐based metabolomics platform has important clinical potential for the early diagnosis and screening of DKD and differential diagnosis of DKD and NDKD in T2DM patients with CKD.

However, there are limitations to this study: (a) although the patients with DKD and diabetes were gender‐matched, there were significant differences in age. And succinic anhydride, 4‐methylbenzaldehyde, and 5‐methylfuran‐2‐carboxylic acid was significantly correlated with age, but not correlated with indicators of DKD progress, suggesting that the difference of those three metabolites is on a level between DKD patients and diabetes patients and might be related to the age, but not DKD itself; further studies are needed to rule out age‐related differences in metabolic profiling. (b) In this study, there were only 20 samples of DKD and 20 samples of diabetes patients, and the sample size was relatively small; only one dataset was used for model training, and there was no validation set or test set to validate the performance of the metabolite panel in identifying DKD and T2DM. (c) All samples in this study were not pathologically confirmed by kidney biopsy, especially those in the DKD group, and NDKD could not be excluded completely. But the patients with the presence of kidney dysfunction before the onset of diabetes was excluded in this investigation. In the future, the sample size will be expanded to further explore the difference of serum metabolic profiling between patients with DKD and patients with diabetes. At the same time, the serum metabolic profiling of NDKD patients will be analyzed to explore the difference between NDKD and DKD and a prospective research to screen metabolite biomarkers for predicting DKD will be conducted.

## Conclusion

In this study we found that LDI‐TOF‐MS‐based metabolomics revealed a metabolic signature in the serum from patients with DKD, which was different from diabetes patients, including butyric acid metabolism, tricarboxylic acid cycle, alanine, aspartic acid, and glutamate metabolism in disorder. l‐kynurenine was correlated with UACR, and l‐serine, pimelic acid, 5‐methylfuran‐2‐carboxylic acid, 4‐methylbenzaldehyde, and dihydrouracil were significantly correlated with eGFR. The metabolite panel had excellent performance in distinguishing patients with diabetes and DKD. LDI‐TOF‐MS‐based metabolomics has the potential for clinical practice in the diagnosis of DKD.

## Conflict of Interest

The authors declare no conflicts of interest.

### Peer review

The peer review history for this article is available at https://www.webofscience.com/api/gateway/wos/peer‐review/10.1002/2211‐5463.13683.

## Author contributions

FQ—literature search, study design, data analysis, writing. LZ—literature search, study design, data interpretation, writing. DZ—data analysis, data interpretation, writing. MY—data collection, data analysis, data interpretation. WZ—data collection, data analysis, data interpretation, writing. JJ—literature search, study design, data interpretation, writing.

## Supporting information


**Fig. S1.** Heatmap of spearman correlation coefficient between metabolic profiling of serum samples from 3 patients with diabetes and 3 patients with DKD in triplicate.

**Fig. S2.** Heatmap of correlation coefficient between 24 differential metabolites and demographic and clinical characteristics. Pearson correlation was used between numerical variables, and polyserial correlation was used between numerical and categorical variables. * p < 0.05, ** p < 0.01 after FDR correction. AOD, age of onset of diabetes; DD, duration of diabetes; BG, blood glucose; TP, total protein; ALB, albumin; mALB, urinary microalbumin; BUN, urea nitrogen; CRE, creatinine.

**Fig. S3.** Heatmap of correlation coefficient between 24 differential metabolites. Pearson correlation coefficient was used to analyze the correlation between metabolites. * p < 0.05, ** p < 0.01. The lower left corner is the result before FDR correction, and the upper right corner is the result after FDR correction.

**Fig. S4.** Heatmap of correlation coefficient between demographic and clinical characteristics. Pearson correlation was used between numerical variables, polyserial correlation was used between numerical and categorical variables, and polychoric correlation was used between categorical variables. * represents p < 0.05, ** represents p < 0.01. The lower left corner is the result before FDR correction, and the upper right corner is the result after FDR correction. AOD, age of onset of diabetes; DD, duration of diabetes; BG, blood glucose; TP, total protein; ALB, albumin; mALB, urinary microalbumin; BUN, urea nitrogen; CRE, creatinine.

## Data Availability

The authors confirm that the data supporting the findings of this study are available within the article (and/or its [Link], [Link], [Link], [Link]).
