# Supplementary figures and images for: Serum metabolomics detected by LDI‐TOF‐MS can be used to distinguish between diabetic patients with and without diabetic kidney disease
Source: FEBS Open Bio. 2023 Aug 11;13(10):1844–58. doi: 10.1002/2211-5463.13683 (PMC10549217; doi:10.1002/2211-5463.13683)

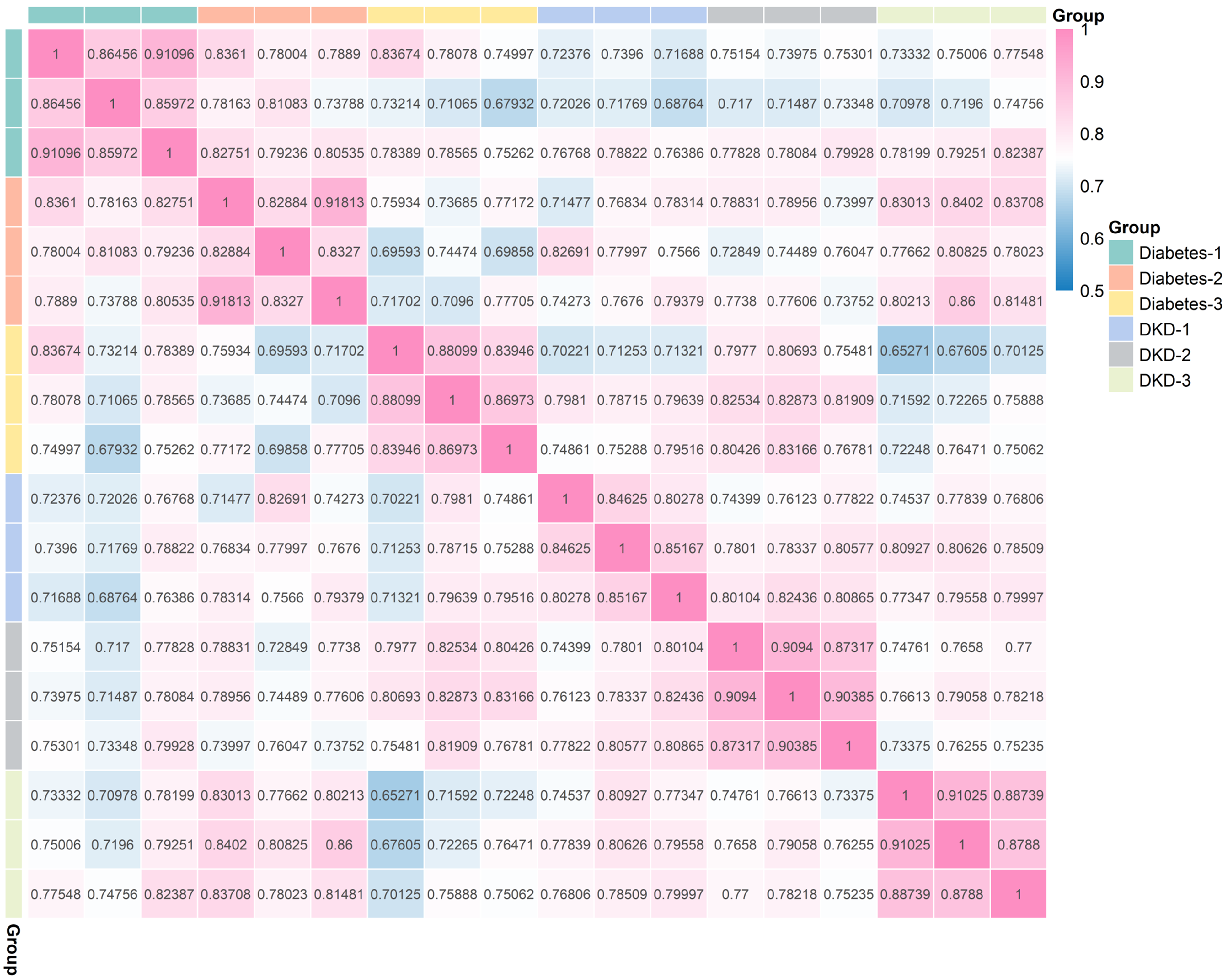

Supplement: Supplementary file 1 — Fig. S1. Heatmap of spearman correlation coefficient between metabolic profiling of serum samples from 3 patients with diabetes and 3 patients with DKD in triplicate. [file FEB4-13-1844-s004.tif]

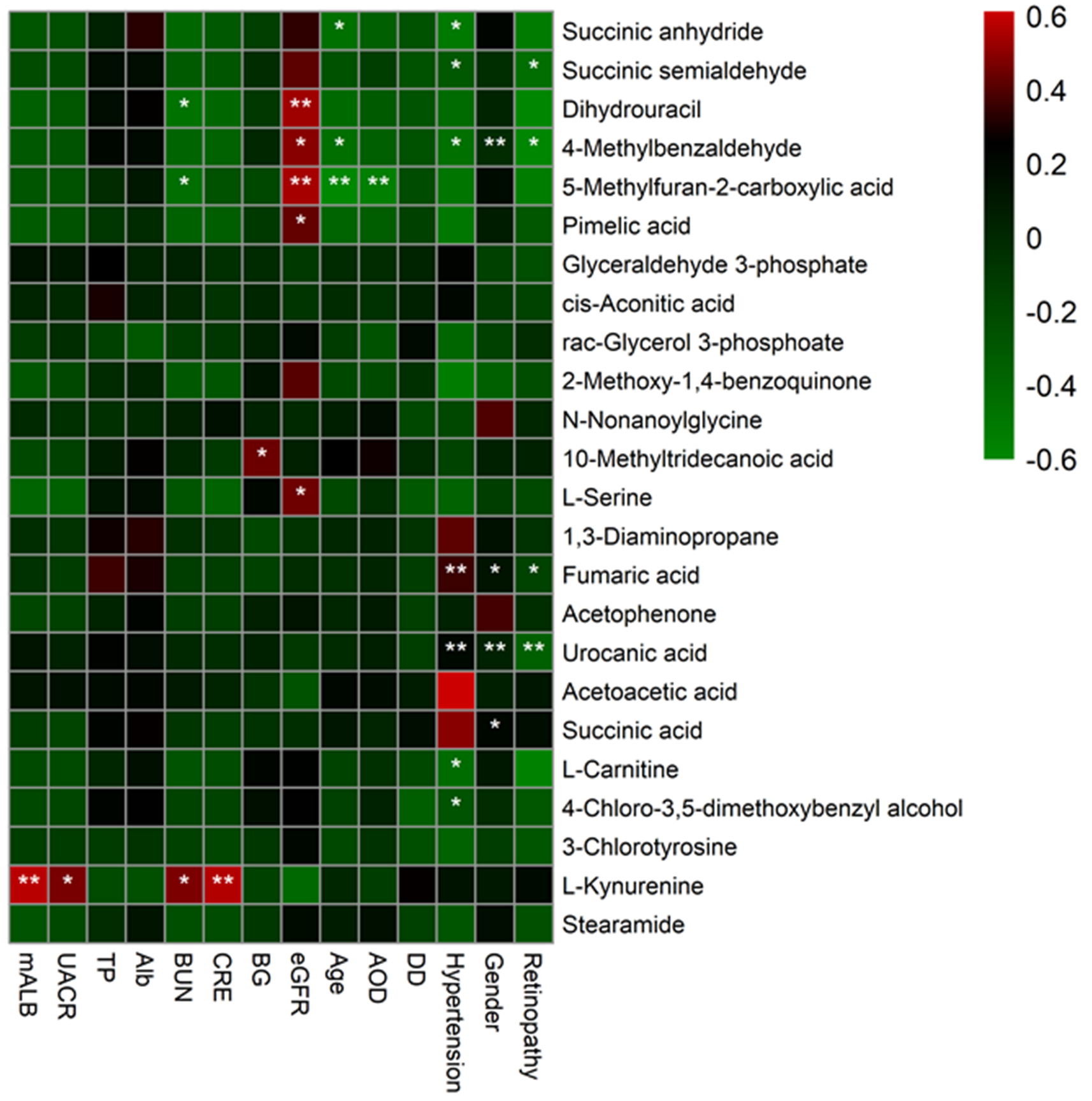

Supplement: Supplementary file 2 — Fig. S2. Heatmap of correlation coefficient between 24 differential metabolites and demographic and clinical characteristics. Pearson correlation was used between numerical variables, and polyserial correlation was used between numerical and categorical variables. * p < 0.05, ** p < 0.01 after FDR correction. AOD, age of onset of diabetes; DD, duration of diabetes; BG, blood glucose; TP, total protein; ALB, albumin; mALB, urinary microalbumin; BUN, urea nitrogen; CRE, creatinine. [file FEB4-13-1844-s001.tif]

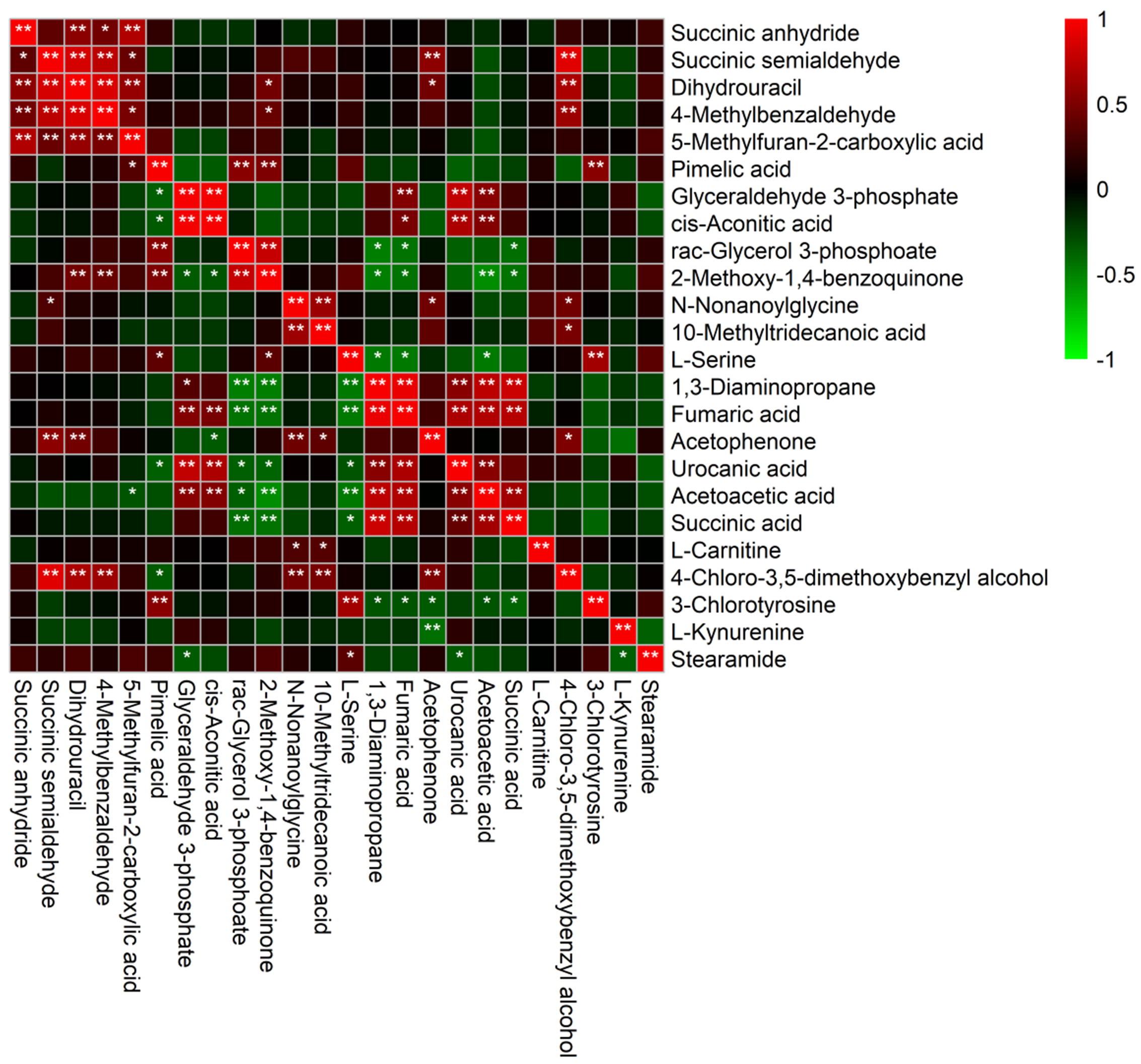

Supplement: Supplementary file 3 — Fig. S3. Heatmap of correlation coefficient between 24 differential metabolites. Pearson correlation coefficient was used to analyze the correlation between metabolites. * p < 0.05, ** p < 0.01. The lower left corner is the result before FDR correction, and the upper right corner is the result after FDR correction. [file FEB4-13-1844-s003.tif]

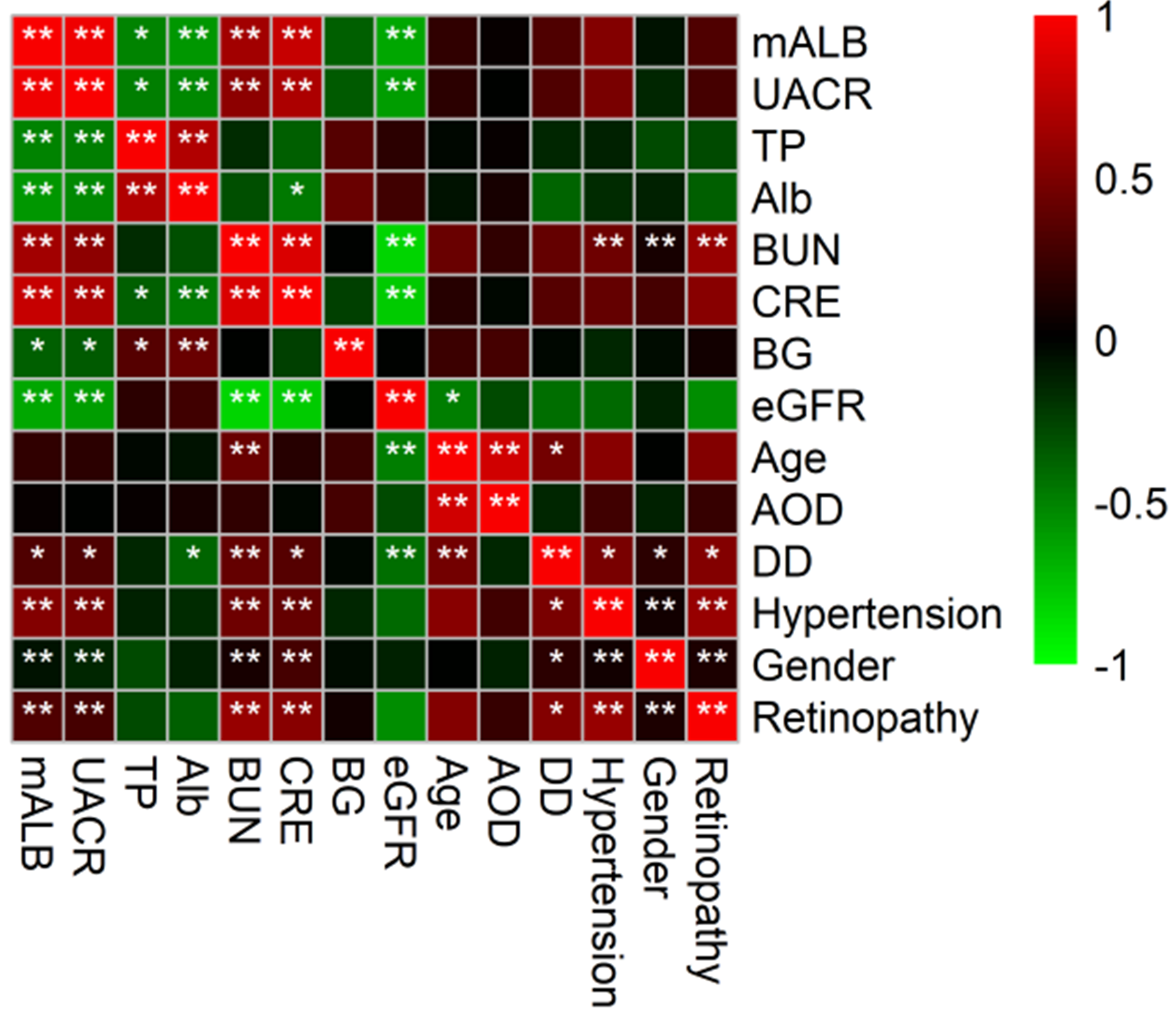

Supplement: Supplementary file 4 — Fig. S4. Heatmap of correlation coefficient between demographic and clinical characteristics. Pearson correlation was used between numerical variables, polyserial correlation was used between numerical and categorical variables, and polychoric correlation was used between categorical variables. * represents p < 0.05, ** represents p < 0.01. The lower left corner is the result before FDR correction, and the upper right corner is the result after FDR correction. AOD, age of onset of diabetes; DD, duration of diabetes; BG, blood glucose; TP, total protein; ALB, albumin; mALB, urinary microalbumin; BUN, urea nitrogen; CRE, creatinine. [file FEB4-13-1844-s002.tif]
